# Supplementary material for: DeepDISE: DNA Binding Site Prediction Using a Deep Learning Method
Source: Int J Mol Sci. 2021 May 24;22(11):5510. doi: 10.3390/ijms22115510 (PMC8197219; doi:10.3390/ijms22115510)
Supplement: Supplementary file 1 [file ijms-22-05510-s001.zip › ijms-1188006-supplementary.pdf]

Supplementary Materials

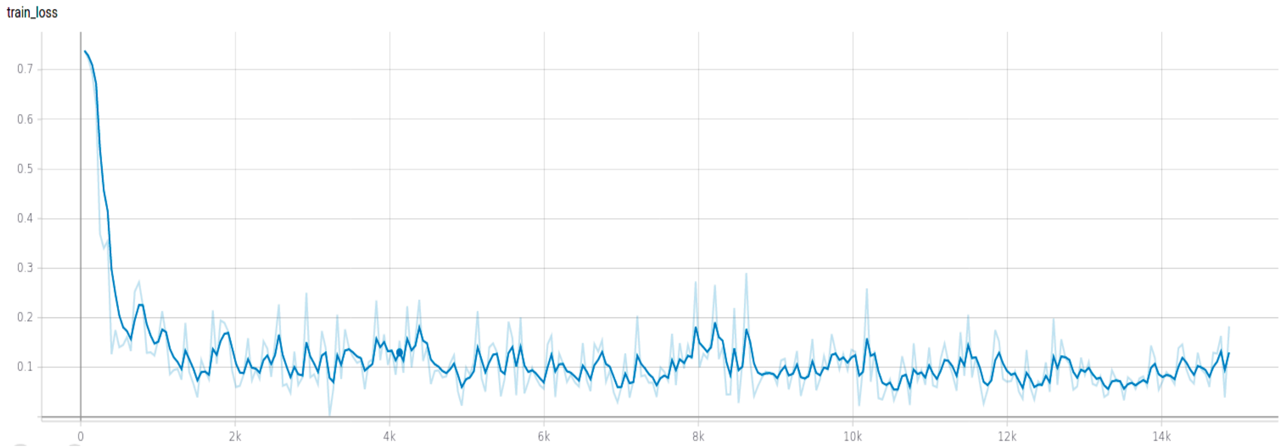

Figure S1. The trajectory of the Loss function of the training dataset

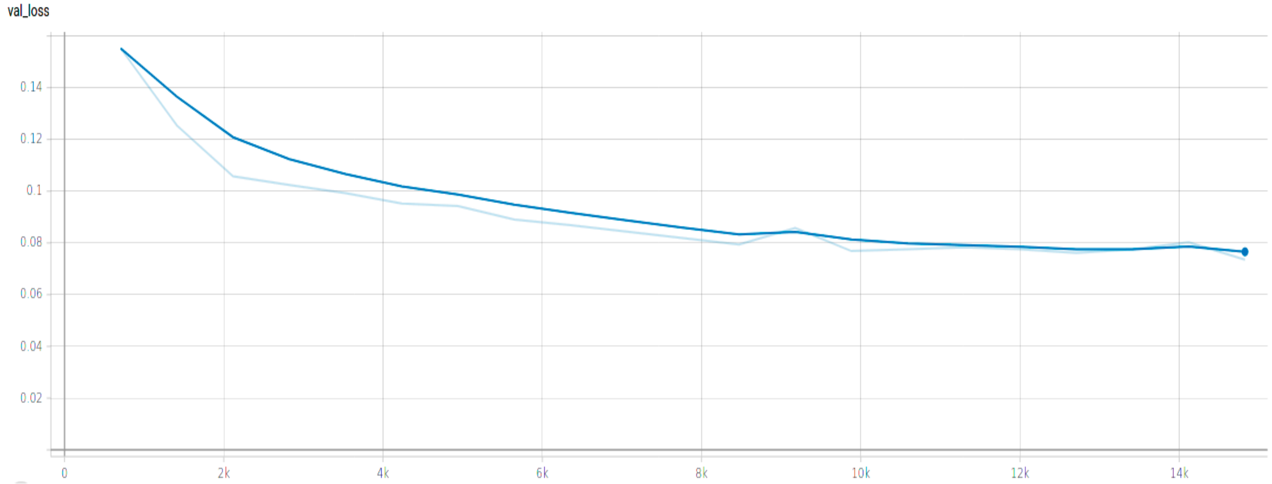

Figure S2. The trajectory of the Loss function of the validation set
